# Functional modifications associated with gastrointestinal tract organogenesis during metamorphosis in Atlantic halibut (*Hippoglossus hippoglossus*)

**DOI:** 10.1186/1471-213X-14-11

**Published:** 2014-02-19

**Authors:** Ana S Gomes, Yuko Kamisaka, Torstein Harboe, Deborah M Power, Ivar Rønnestad

**Affiliations:** 1 Department of Biology, University of Bergen, Po. Box 7803, NO-5020 Bergen, Norway; 2 Institute of Marine Research, Austevoll Aquaculture Research Station, NO-5392 Storebø, Norway; 3 Comparative and Molecular Endocrinology Group, Centre for Marine Sciences (CCMAR), University of Algarve, Campus de Gambelas, 8005-139 Faro, Portugal

**Keywords:** Atlantic halibut, Gastric proton pump, Gastrointestinal tract, Ghrelin, Motility, Na^+^/K^+^-ATPase, Pepsinogen, Ontogeny, pH, Stomach

## Abstract

**Background:**

Flatfish metamorphosis is a hormone regulated post-embryonic developmental event that transforms a symmetric larva into an asymmetric juvenile. In altricial-gastric teleost fish, differentiation of the stomach takes place after the onset of first feeding, and during metamorphosis dramatic molecular and morphological modifications of the gastrointestinal (GI-) tract occur. Here we present the functional ontogeny of the developing GI-tract from an integrative perspective in the pleuronectiforme Atlantic halibut, and test the hypothesis that the multiple functions of the teleost stomach develop synchronously during metamorphosis.

**Results:**

Onset of gastric function was determined with several approaches (anatomical, biochemical, molecular and *in vivo* observations). *In vivo* pH analysis in the GI-tract lumen combined with quantitative PCR (qPCR) of α and β subunits of the gastric proton pump (*H*^*+*^*/K*^*+*^*-ATPase*) and *pepsinogen A2* indicated that gastric proteolytic capacity is established during the climax of metamorphosis. Transcript abundance of *ghrelin*, a putative orexigenic signalling molecule produced in the developing stomach, correlated (p < 0.05) with the emergence of gastric proteolytic activity, suggesting that the stomach’s role in appetite regulation occurs simultaneously with the establishment of proteolytic function. A 3D models series of the GI-tract development indicated a functional pyloric sphincter prior to first feeding. Observations of fed larvae *in vivo* confirmed that stomach reservoir function was established before metamorphosis, and was thus independent of this event. Mechanical breakdown of food and transportation of chyme through the GI-tract was observed *in vivo* and resulted from phasic and propagating contractions established well before metamorphosis. The number of contractions in the midgut decreased at metamorphic climax synchronously with establishment of the stomach’s proteolytic capacity and its increased peristaltic activity. Putative osmoregulatory competence of the GI-tract, inferred by abundance of *Na*^*+*^*/K*^*+*^*-ATPase α* transcripts, was already established at the onset of exogenous feeding and was unmodified by metamorphosis.

**Conclusions:**

The functional specialization of the GI-tract was not exclusive to metamorphosis, and its osmoregulatory capacity and reservoir function were established before first feeding. Nonetheless, acid production and the proteolytic capacity of the stomach coincided with metamorphic climax, and also marked the onset of the stomach’s involvement in appetite regulation via ghrelin.

## Background

The divergent gastrointestinal (GI-) tract morphology and feeding strategies between larval and adult phases are adaptations to fundamentally different habitats and food resources [1]. The post-embryonic maturation of the digestive system is a key event in the life history of vertebrates and essential for survival. Thyroid hormone (TH) driven metamorphosis plays a crucial role in the functional maturation of the GI-tract and in shaping its morphology to the adult form [2,3]. Remodelling of the GI-tract from larva to adult has been extensively studied in *Xenopus*[2,4]. In this organism, the intestine is transformed under the influence of THs from a long coiled tube into a complex organ with a differentiated stomach and small intestine [5,6]. This involves epithelial and mesenchymal proliferation, smooth muscle thickening and the formation of intestinal folds. Several studies have described the cellular mechanisms responsible for this remodelling in amphibians [7,8], yet little is known about their impact on tissue function in vertebrates, particularly the multiple functions integrated in the stomach.

A striking feature of vertebrate metamorphosis is the organogenesis of the stomach. In early developmental stages of fishes and anurans the stomach is often absent and part of its function may be carried out by the intestine. The main roles of the vertebrate stomach are storage of ingested food, secretion of hydrochloric acid (HCl) and pepsinogen, and mechanical breakdown and mixing of food with gastric secretions [1,9]. Thus, in larvae of altricial-gastric species, such as the Atlantic halibut, the absence of a stomach limits the ability to digest dietary protein when exogenous feeding is initiated [10-14]. This is one of the reasons why most studies of GI-tract development during metamorphosis have focused on stomach development and consider the appearance of gastric glands as an adequate indicator of a fully developed stomach [15,16]. However, it has become clear that the histological identification of gastric glands does not indicate that the stomach is fully functional. Hence, the stomach’s proteolytic function is best indicated by pepsin activity [11,17] and *pepsinogen* content [18]. To better understand the efficiency of digestive processing during fish ontogeny, several studies compared expression profiles of *pepsinogen* and the gastric proton pump (*H*^*+*^*/K*^*+*^*-ATPase*), localized in the HCl secreting oxynticopeptic cells [19-22]. Murray et al. [23] have used histology and *pepsinogen* transcript analysis to study the ontogeny of the stomach in Atlantic halibut and showed that the appearance at 66 days post-hatching (dph) of gastric glands preceded expression of *pepsinogens A1* and *A2* transcripts at 80 dph. However, the impact of metamorphosis on other important functions of the stomach or GI-tract development in general has largely been overlooked in flatfish.

In addition to acid production and proteolysis the vertebrate stomach also has reservoir functions. After ingestion, the stomach stores and predigests food, then delivers the chyme to the midgut for further digestion and subsequent nutrient absorption [9]. The storage function of the stomach relieves juvenile and adult fish from the need to constantly feed like the stomachless larval stages. Establishment of the stomach as a reservoir requires functional sphincters (esophagus and pylorus) and well developed neural and smooth muscle layers. The mechanical mixing and transport of food through the GI-tract is achieved by specific motility patterns and by matching peristalsis with the release of digestive enzymes. This process plays a central role in effective food processing (see review, [24]), though very few studies have targeted GI-tract movements in fish larvae. The advantage of using fish larvae, such as Atlantic halibut, is their optic transparency that is maintained until metamorphosis. This permits direct visual observations of the GI-tract and its motility patterns in live animals. Pittman et al. [25] reported peristaltic contractions in Atlantic halibut larvae, in the anterior intestine at 35 dph. In juvenile Atlantic halibut GI-tract both anterograde (propagating in the anal direction) and retrograde (propagating in the oral direction) contraction waves were described [26], and identical patterns were also observed in embryos and larvae of the stomachless zebrafish (*Danio rerio*) [27].

The stomach produces hormones involved both in the regulation of appetite and digestion. Ghrelin is an example of a hormone that is mainly produced in the stomach and acts as a stimulator of food intake [28,29]. In mammals, ghrelin has also been suggested to stimulate gastric acid secretion and motility [30,31]. The function of ghrelin in fish larvae is still poorly described, but it has been proposed as an indicator of the stomach’s involvement in appetite regulation in developing fish [32]. In Atlantic halibut, *ghrelin* gene expression increases during the climax of metamorphosis, coinciding with stomach development [33]. Ghrelin is abundant in the developing gastric glands and several osmoregulatory tissues. Additionally, its co-expression with *Na*^*+*^*/K*^*+*^*-ATPase* suggests a putative role in hydromineral balance [34]. Yet, the role of ghrelin in appetite regulation, motility and osmoregulation is unknown, as well as its link to the proteolytic and reservoir function of the stomach in Atlantic halibut during metamorphosis.

This study aims to establish the impact of the agastric-gastric transition on the functional role of the post-embryonic GI-tract remodelling that occurs during metamorphosis in Atlantic halibut, a flatfish species of high commercial interest for the Northern European and North American aquaculture industry. To map the changes in GI-tract development and establish events linked to TH-driven metamorphosis we constructed a series of 3D models of the morphological and spatial organization of the digestive organs in representative developmental stages. We tested the hypothesis that the development of the multiple stomach functions is synchronous and linked to its physical appearance at metamorphosis. The proteolytic function of the stomach was studied using *in vivo* pH analyses combined with expression profiles of the specific gene markers *H*^*+*^*/K*^*+*^*-ATPase α* and *β subunit* and *pepsinogen A2* using quantitative PCR (qPCR). Stomach filling and reservoir function were assessed by *in vivo* visual studies of the transparent larvae at prometamorphosis and climax of metamorphosis. The putative role of a fully functional stomach in appetite regulation was assessed by measuring *ghrelin* transcript abundance. The establishment of GI-tract motility patterns during development was determined by *in vivo* observations and the involvement of the GI-tract in osmoregulation was assessed by measuring the abundance of *Na*^*+*^*/K*^*+*^*-ATPase α* subunit transcripts.

## Results

### 3D reconstruction of digestive organs

**Figure 1:**
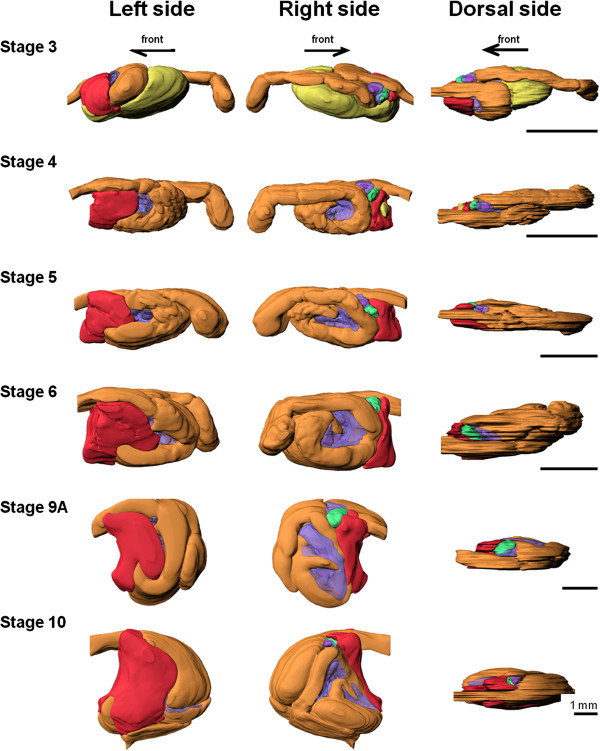
**Ontogeny of the digestive organs in Atlantic halibut larvae.** 3D models were reconstructed from serial histological sections using Imaris software. The digestive organs are shown from three angles; left, right and dorsal side. *Arrows* indicate the anterior direction (mouth). *Orange* outer layer of GI-tract, *red* liver, *green* gallbladder, *purple* pancreas, *pink* islet of Langerhans, *yellow* yolk-sac. Transparent colour is used for exocrine pancreas (*purple*) in order to show islets of Langerhans (*pink*) and gallbladder (*green*).

3D models of the morphology of the digestive system during development were reconstructed from a series of histological sections. Location and size of the GI-tract and its associated organs, such as liver, endocrine and exocrine pancreas, and gallbladder, were observed from stage 3 (prior to first feeding) until the post-metamorphic stage 10 (Figure 1).

**Figure 2:**
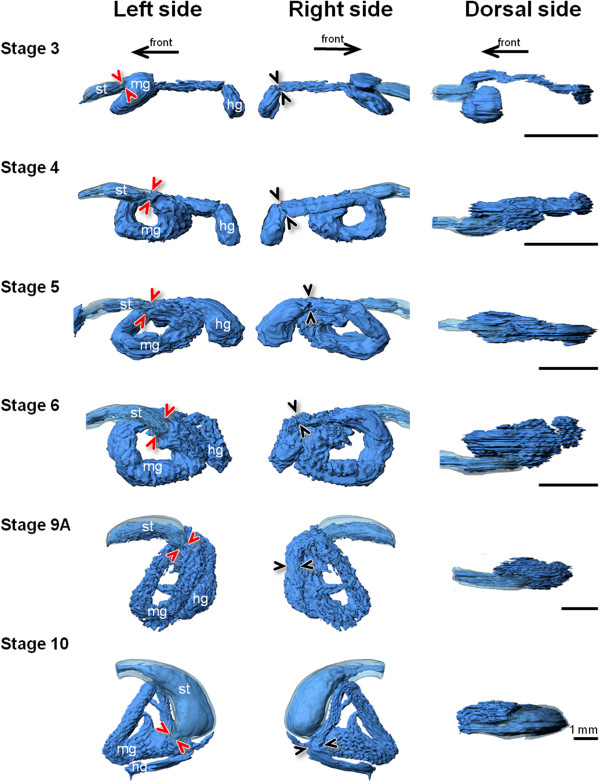
**Ontogeny of presumptive stomach (*transparent light blue*) and GI-tract lumen (*blue*) in Atlantic halibut larvae.** 3D models were reconstructed from serial histological sections using Imaris software. GI-tract lumen is represented by the inner layer (facing the lumen) of the GI-tract. The GI-tract is seen from three angles; left, right, and dorsal side. *Arrows* indicate the anterior direction (mouth). *Arrow heads* point to position of sphincters (red: pyloric sphincter; black: ileorectal sphincter). *st* presumptive stomach/stomach, *mg* midgut, *hg* hindgut.

**Figure 3:**
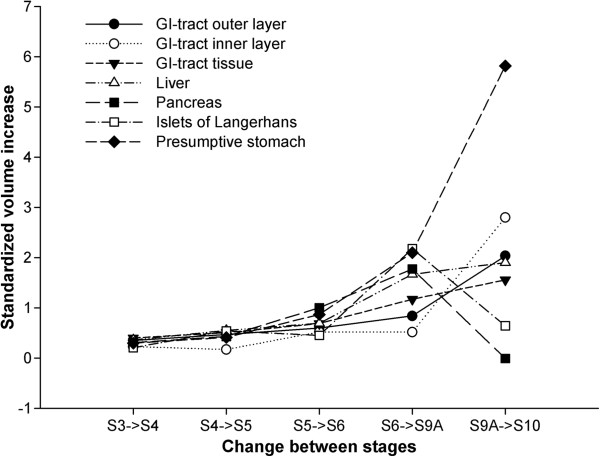
**Standardized volume increase of digestive organs between stage (S) 3 to 10 of Atlantic halibut.** The volume increase was normalized to the overall mean of volume increase between stages for each tissue (for detailed explanation, see Additional file 2).

The GI-tract includes a narrow foregut (esophagus and presumptive stomach/stomach), midgut, and a short hindgut (rectum) (Figure 2). The anterior region of the midgut, just after the pyloric sphincter (PS), was larger in diameter, i.e. more voluminous, compared to the rest of the midgut. This feature was maintained during GI-tract ontogeny (Figures 1 and 2). Both PS (which separates the presumptive stomach from the anterior midgut) and ileorectal sphincter (which separates midgut and hindgut) were identified from stage 3 onwards (Figures 1 and 2). Pyloric caeca became evident as projections from the most anterior part of the midgut at stage 6 (Figures 1 and 2). The stomach was well-differentiated at stage 10 and the gastric glands were visible on histological sections (Additional file 1). The luminal volume of the GI-tract increased during development, particularly in the two last stages analysed (stages 9A and 10) (Figure 3, Table 1 and Additional file 2). The stomach volume from 9A to 10 increased from 415 to 4933 nl, respectively and corresponded to an 11 fold increment (Table 1).

**Table 1:** **GI-tract organ volume (nl) and surface area (10**^**6**^ **μm**^**2**^**)**

	**Stage 3**	**Stage 4**	**Stage 5**	**Stage 6**	**Stage 9A**	**Stage 10**
GI-tract outer layer (nl)	157.42	261.03	490.65	1038.48	2670.15	12855.10
GI-tract outer layer (10^6^ μm^2^)	3.59	5.12	6.74	10.07	20.53	63.38
GI-tract inner layer (nl)	78.40	136.84	266.55	525.89	1034.10	6451.16
GI-tract inner layer (10^6^ μm^2^)	2.83	4.73	9.60	16.34	50.45	54.76
GI-tract tissue volume^a^ (nl)	79.02	124.19	224.10	512.59	1636.05	6403.94
Liver (nl)	35.24	48.82	98.18	225.23	928.25	4232.77
Pancreas (nl)	13.79	21.42	37.91	109.08	471.25	463.79
Islets of langerhans (nl)	0.50	0.46	0.57	1.05	5.33	11.73
Presumptive stomach (nl)	15.51	27.29	32.06	84.09	414.54	4932.67

The values were calculated from the 3D models using Imaris MeasurementsPro.

^a^GI-tract tissue volume = GI-tract outer layer - GI-tract inner layer.

The liver was positioned under the foregut and anterior to the ascending loop of the midgut (Figure 1) and its volume steadily increased during development (Figure 3 and Table 1). The exocrine pancreas was observed between the presumptive stomach and the anterior part of the midgut at stage 3 and it surrounded this midgut area throughout ontogeny (Figure 1). In the endocrine pancreas, a clearly distinguishable islet of Langerhans was observed close to the gallbladder at stage 3 (Figure 1). In contrast to the other digestive organs, the increment in the normalized volume of endocrine and exocrine pancreas was low and negative, respectively, between stages 9A and 10 (Figure 3 and Table 1). The yolk-sac, positioned under the GI-tract at stage 3, decreased in size after the initiation of exogenous feeding and a small vestige remained besides the liver at stage 4 (6 days post first feeding, dpff). The gallbladder was observed on the right-hand side between the exocrine pancreas and the liver, and maintained this position in all the developmental stages analysed (Figure 1). The pancreatic duct and the bile duct opened next to each other into the lumen at the median plane of the anterior midgut, just after the PS (data not shown).

### Cloning and phylogenetic characterization of pepsinogen A2, ghrelin, gastric proton pump subunits and Na^+^/K^+^-ATPase subunit α

The complete coding sequence (CDS) of Atlantic halibut *pepsinogen A2* was 1128 bp and was submitted to GenBank under accession no. KF184647 (Additional file 3: C). The amino acid (AA) sequence of pepsinogen is relatively well-conserved among teleost fish and, as expected, more variable when compared to other vertebrate pepsinogens. For instance, halibut pepsinogen A2 shared respectively 88% and 64% AA sequence identity with winter flounder (*Pseudopleuronectes americanus*) pepsinogen A form IIb and IIa, but only 52% and 48% identity with homologues from *Xenopus laevis* and human, respectively (data not shown).

The cDNA fragments cloned for Atlantic halibut *H*^*+*^*/K*^*+*^*-ATPase α subunit* (911 bp) and *Na*^*+*^*/K*^*+*^*-ATPase α subunit* (714 bp) were deposited in GenBank with the accession numbers KF184648 and KF184650, respectively (Additional file 3: B, D). The CDS for *H*^*+*^*/K*^*+*^*-ATPase β subunit* of 874 bp was cloned and submitted to GenBank with the accession no. KF184649 (Additional file 3: A). Phylogenetic analysis of the α subunit of the gastric proton pump and Na^+^/K^+^-ATPase, and vertebrate homologues (Additional file 4) generated two major clades, one corresponding to H^+^/K^+^-ATPase and the other to Na^+^/K^+^-ATPase. Phylogenetic analysis of the β subunit (Additional file 5) generated a tree with two major clades that shared the same general topology as the phylotree for the α subunit with the H^+^/K^+^-ATPase and Na^+^/K^+^-ATPase clustered independently.

Atlantic halibut H^+^/K^+^-ATPase α subunit clustered most closely with teleost homologues, with which it shared 94% AA sequence identity, and increased to 98% identity with winter flounder and Atlantic cod (*Gadus morhua*). Lower AA sequence identity (72%) was found when Atlantic halibut H^+^/K^+^-ATPase α subunit was compared to Atlantic halibut Na^+^/K^+^-ATPase α subunit (70%) and to other vertebrate counterparts (72%). The Atlantic halibut Na^+^/K^+^-ATPase α subunit clustered with an Antarctic eelpout (*Pachycara brachycephalum*) homologue (98%) and shared approximately 88% AA identity with other teleost gene homologues. H^+^/K^+^-ATPase β subunit clustered as expected within the teleost clade (overall identity about 80%) and shared rather low identity with its human homologue (50%). Atlantic halibut H^+^/K^+^-ATPase β subunit did not share more than 39% AA sequence identity with the Atlantic halibut Na^+^/K^+^-ATPase β subunit.

### Ontogenetic expression pattern and correlation analysis

**Figure 4:**
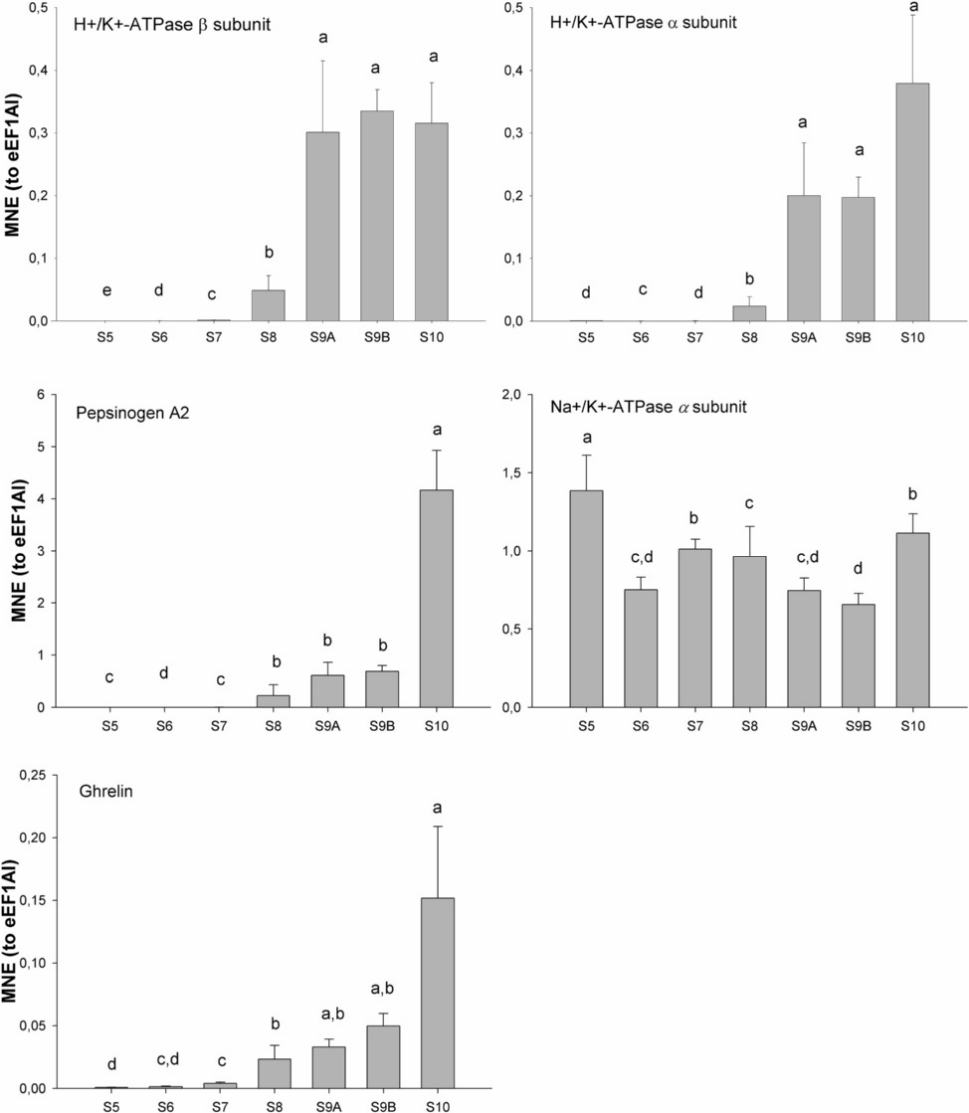
**Mean normalized expression patterns of the indicated mRNA transcripts of individual larvae (stage 5–10).** Results for *pepsinogen A2* precursor, gastric *H*^*+*^*/K*^*+*^*-ATPase subunit α* and *β*, *Na*^*+*^*/K*^*+*^*-ATPase subunit α* and *ghrelin* mRNA transcripts are shown as mean ± SEM of the normalized expression (using the reference gene eEF1A1). Mean values with different letters are significantly different (One Way ANOVA, p < 0.05).

**Figure 5:**
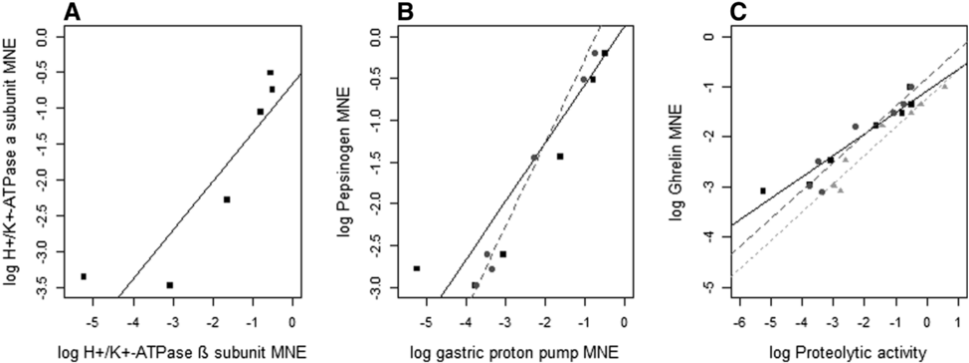
**Linear regressions estimated from correlation analyses between stomach specific gene markers during Atlantic halibut ontogeny.** Linear regression models were fitted to log-transformed mean expression values (MNE) of **A)** gastric *H*^*+*^*/K*^*+*^*-ATPase α subunit* and *β subunit*; **B)***pepsinogen A2* and *H*^*+*^*/K*^*+*^*-ATPase β subunit* (black squares and solid line) as well as *H*^*+*^*/K*^*+*^*-ATPase α subunit* (grey dots and dashed line); **C)***ghrelin* and *H*^*+*^*/K*^*+*^*-ATPase β subunit* (black squares and solid line), *H*^*+*^*/K*^*+*^*-ATPase α subunit* (grey dots and dashed line) as well as *pepsinogen A2* (light grey triangles and dotted line). All correlations are significant (p < 0.05). The log-transformed mean of MNE per stage (5 to10) was taken from six individuals.

The developmental expression profiles of *pepsinogen A2*, *H*^*+*^*/K*^*+*^*-ATPase α* and *β subunits*, *Na*^*+*^*/K*^*+*^*-ATPase α subunit* and *ghrelin* were analysed by qPCR in the GI-tract of individual Atlantic halibut larvae (Figure 4). The gene expression of both gastric proton pump subunits were significantly (p < 0.05; adjusted R^2^: 0.773) correlated (Figure 5) and had parallel expression patterns, with a sharp and significant (p < 0.05) increase at climax and in post-metamorphic stages (Figure 4). *Pepsinogen A2* was significantly (p < 0.05) correlated with the expression profile of the gastric proton pump α (adjusted R^2^: 0.9738) and β (adjusted R^2^: 0.7963) subunits (Figure 5). A significant (p < 0.05) increase during stage 8 was observed for *pepsinogen A2* and its expression peaked in the post-metamorphic stage. *Ghrelin* mRNA transcript abundance increased gradually and significantly (p<0.05) during the proclimax/climax of metamorphosis, and attained a maximum in the post-metamorphic stage (Figure 4). Moreover, ghrelin transcript abundance and proteolytic activity during GI-tract ontogeny were significantly correlated (p < 0.05; adjusted R^2^: 0.9342, 0.8852, 0.9252 for pepsinogen A2, gastric proton pump α and β subunits, respectively; see Figure 5). Expression of *Na*^*+*^*/K*^*+*^*-ATPase α subunit* mRNA was detected in all developmental stages, with significantly (p < 0.05) more transcripts at stage 5.

### Estimation of pH in the lumen of stomach and detection of acid production

**Figure 6:**
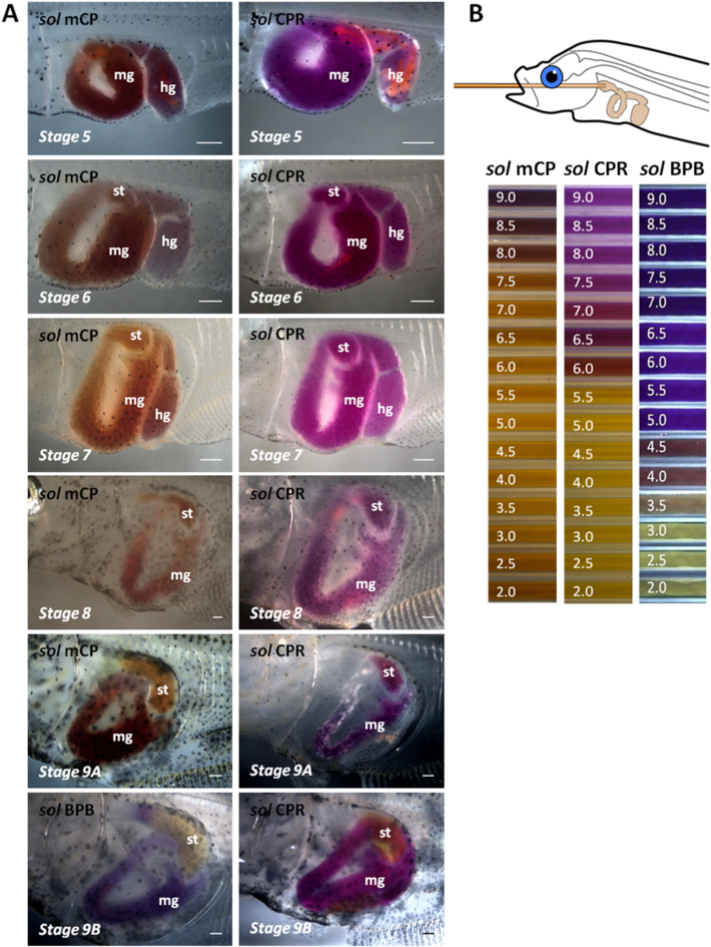
**pH changes in the GI-tract of Atlantic halibut larvae during development. Panel A:** results of tube feeding pH indicator solutions into larva from premetamorphosis (stage 5) to climax metamorphosis (stage 9A and 9B). pH *sol* mCP contained 0.1% M-Cresol purple, *sol* CPR consisted of 0.1% Chlorphenol Red and pH *sol* BPB had 1% of Bromophenol blue. st: stomach/presumptive stomach; mg: midgut; hg: hindgut. Scale bar = 0.5 mm. **Panel B:** standards immersed in water and photographed with similar light condition as larvae under the dissecting microscope.

The pH assessment in the lumen of the stomach and midgut/hindgut during post-embryonic development was based on the colour observed after the injection of pH indicator solutions (Figure 6 and Table 2). The pH in the midgut/hindgut remained alkaline (above pH 8) in all the developmental stages analysed (stage 5 to 9B). The presumptive stomach also had an alkaline pH with values above 7.5 until stage 8. Gradual acidification was observed in the stages corresponding to the climax of metamorphosis. Transition from an alkaline to an acidic pH in the stomach lumen was evident at stage 9A, when the injected *sol* CPR remained purple but the *sol* mCP gave a yellow coloration (pH6.5 – 7.5). The lumen of the stomach was clearly in the acidic range (pH < 3.5) at stage 9B, as revealed by the yellow colour in the stomach following administration of both CPR and BPB solutions.

**Table 2:** pH changes in the GI-tract of Atlantic halibut larvae at different developmental stages

**Stage**	**Stomach**	**Midgut/Hindgut**
**5**	>7.5	>8.0
**6**	>7.5	>8.0
**7**	>7.5	>8.0
**8**	>7.5	>8.0
**9A**	6.5 – 7.5	>8.0
**9B**	<3.5	>6.5

The presented pH values are based on visual observations of colour changes after the administration of three pH indicator solutions.

### Analysis of GI-tract motility

**Figure 7:**
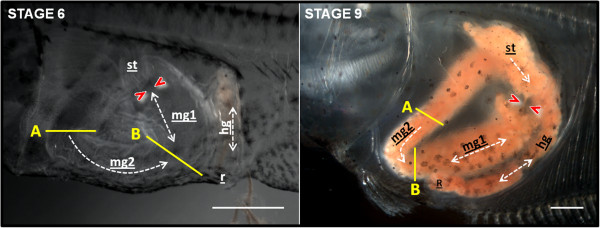
**GI-tract motility in Atlantic halibut larvae at stage 6 and 9.** Retrograde and anterograde wave movements (propagating contractions) are indicated by a dashed line. These waves occur from area A to B; and from pyloric sphincter (represented by two red arrows*) to area B and *vice-versa*. A and B represent the main areas where phasic contractions occur. st: stomach/presumptive stomach; mg1: midgut region 1; mg2: midgut region 2; hg: hindgut; r: rectal area. Scale bar = 1 mm.

Spontaneous propagating contractions were observed in the GI-tract at prometamorphosis (stage 6; 25 dpff) and climax of metamorphosis (stage 9A/B; 49 dpff) (Figure 7). Due to considerable individual variation, number and frequency of contractions could not be grouped and are presented for each individual analysed (Table 3 and Additional file 6). Two types of contractions were observed in the midgut region 1 (mg1; after the PS, descending part of the loop) and 2 (mg2): phasic and propagating waves (Additional file 7). The propagating contractions observed in mg2 were retrograde waves that originated in area “*A*” and moved towards the mouth. However, in mg1 most of the propagating contractions originated just under the PS and were anterograde waves that moved in an anal direction. Motility activity in both midgut regions was detected at stage 6 with a frequency ranging from 0.31 to 3.77 min^-1^, depending on the individual and type of contraction. At stage 9, relatively few spontaneous contractions of short duration were observed in the midgut. During the climax of metamorphosis, contractions in the stomach were registered in all individuals, in contrast to stage 6 when motility in the presumptive stomach was only observed in one larva. The rectal contraction (or defecation reflex) was a mixture of retro- and anterograde contractions and were observed in both stages 6 and 9 with similar frequencies in most of the individuals analysed.

**Table 3:** GI-tract motility patterns - quantification

		**Stomach**		**Midgut 1**				**Midgut 2**				**Hindgut**		**Rectal area**	
				***Propagating***		***Phasic***		***Propagating***		***Phasic***					
	**Larva**	***n***	**Frequency (min**^**-1**^**)**	***n***	**Frequency (min**^**-1**^**)**	***n***	**Frequency (min**^**-1**^**)**	***n***	**Frequency (min**^**-1**^**)**	***n***	**Frequency (min**^**-1**^**)**	***n***	**Frequency (min**^**-1**^**)**	***n***	**Frequency (min**^**-1**^**)**
**Stage 6**	1	-	-	14	2.31	50	1.69	57	1.84	79	3.54	13	1.46	161	5.24
	2	-	-	26	1.81	-	-	95	3.77	12	0.44	15	0.54	3	0.22
	3	17	0.56	5	0.31	4	0.24	28	1.66	77	3.01	-	-	96	3.22
	4*	-	-	-	-	-	-	-	-	-	-	43	2.25	25	1.69
**Stage 9**	1	11	2.37	-	-	-	-	-	-	-	-	1	-	11	1.44
	2	10	0.90	-	-	-	-	-	-	-	-	-	-	11	0.41
	3	4	0.44	-	-	-	-	9	3.83	-	-	-	-	13	0.46
	4	4	0.56	-	-	3	1.13	-	-	-	-	12	1.12	24	0.81

Propagating and phasic contractions are stated for midgut 1 and midgut 2 regions. Frequency is the number of contractions registered (n) per min.

*Not possible to quantify phasic and propagating wave contractions. The affected GI-tract segments were constantly (tonic) contracted during the whole observation period (see Additional file 7).

## Discussion

In all altricial-gastric species, the GI-tract undergoes dramatic remodelling during TH-driven metamorphosis. The GI-tract changes from a simple tubular form into a more complex folded structure. At the same time the stomach becomes a distinct compartment and continues to acquire its multiple functions through metamorphosis. There are surprisingly few studies examining and integrating the anatomical and functional changes in the GI-tract associated with this TH-driven event. In amphibians such as *Xenopus*, it is well established that THs induce GI-tract remodelling leading to intestinal shortening and the development of crypts and villi [35-37]. The remodelling of the *Xenopus* digestive tract is a consequence of changes in TH regulated genes, including *sonic hedgehog*/*bone morphogenetic protein-4* (*Shh*/*BMP-4*) and *Tolloid*/*BMP-1*[38-40]. However, in contrast to amphibians, few studies have linked GI-tract remodelling with TH-driven metamorphosis in teleost fish, in particular the pleuronectiformes. The present study used a series of 3D models to reconstruct the ontogeny of Atlantic halibut GI-tract morphology and its volume changes during larval development. Using molecular and functional analysis, the modification of GI-tract function from the premetamorphic stage 5 until the end of metamorphosis (stage 10) was established. Insight was obtained into the way in which post-embryonic differentiation of the GI-tract and the growth of a new organ, the stomach, affects the function of the whole digestive system. Moreover, the results can be extrapolated to other altricial-gastric teleosts to further understand how functional remodelling of the digestive system affects feeding behaviour.

Our 3D models of the digestive organs showed a general trend of volume increase during Atlantic halibut larvae development. During metamorphic climax (stage 9), when THs (T3 and T4) levels were high [41], the Atlantic halibut GI-tract tissue volume increased almost four fold, and the stomach volume increased more than 11-fold. The peak in THs in stage 9 Atlantic halibut coincided with maximal GI-tract remodelling and the changes were reminiscent of what occurs in *Xenopus* (for review, see [2,8,42]). In contrast to the GI-tract and liver, the exocrine and endocrine pancreas appeared to have no growth or only a two fold increase in size, respectively, between the two oldest stages analysed (stage 9A to stage 10). A similar tendency was observed for the pancreas in Atlantic cod during stomach differentiation [43]. The authors hypothesized that this trend may be related to the importance of the pancreatic digestive enzymes in early agastric stages, particularly trypsin [44]. The similarity of the changes in the pancreas and stomach of Atlantic halibut and cod gives credence to the hypothesis, but experimental proof is still required.

The correlation between TH level increments and stomach development during metamorphosis has previously been documented for the pleuronectiformes, Japanese flounder (*Paralichthys olivaceus*) and summer flounder (*Paralichthys dentatus*) [18,45]. In these flatfish, the development of the gastric glands started during pre-metamorphosis, but *pepsinogen* was only detected after metamorphosis. In Atlantic halibut, the gastric glands appeared prior to the TH peak and *pepsinogen* transcripts were detected before metamorphic climax [23]. However, the results were dependent on the method used: with RT-PCR *pepsinogen A2* expression was observed at 80 dph (stage 8) and with *in situ* hybridization (ISH) at 87 dph (stage 9A). Murray et al. [23] suggested that *pepsinogen* expression in Atlantic halibut can only occur when the gastric glands are completely developed. In the present study, *pepsinogen* transcripts were detected in Atlantic halibut GI-tract before the metamorphic climax, suggesting that the proteolytic capacity of the presumptive stomach was triggered by the rise in THs at the start of metamorphosis. Nonetheless, it remains to be demonstrated that *pepsinogen* transcript abundance correlates with proteolytic activity. Future studies discriminating between pepsinogen and pepsin activity will be needed to clarify this issue.

Acid secretion in the stomach requires the gastric H^+^/K^+^-ATPase, an α,β-heterodimeric enzyme, which exchanges a proton with potassium using energy from ATP to generate HCl [46-48]. The α subunit of the enzyme contains the catalytic site and the β subunit is required for proper maturation and targeting of the enzyme to the apical membrane [48]. In the present study the expression of α and β subunit transcripts of gastric H^+^/K^+^-ATPase were synchronous and correlated, as previously reported in mammalian stomach development (e.g. mouse: [49]). In Atlantic halibut the expression of *pepsinogen A2* and both gastric proton pump subunits were also correlated, and similar observations have been made during larval development of the winter flounder, red porgy (*Pagrus pagrus*) and yellow catfish (*Pelteobagrus fulvidraco*), [19-21,50,51]. The synchronous expression of these genes was proposed to be a physiological strategy to promote quick conversion of *pepsinogen* into pepsin [52]. However, it remains to be established if the mechanism for the release of both enzymes from the oxynticopeptic cells is the same or occurs independently [22].

The identification of *pepsinogen* and gastric *H*^*+*^*/K*^*+*^*-ATPase* transcripts or protein indicate the stomachs proteolytic potential but not its actual activity. For this reason in the present study, *in vivo* pH analysis was carried out and revealed the increase in *H*^*+*^*/K*^*+*^*-ATPase α* and *β subunit* transcripts occurred simultaneously with increasing acidic capacity in Atlantic halibut larvae during the climax of metamorphosis (stage 9A and 9B), when TH levels rise. From the first slight acidification observed in the lumen of the stomach during stage 9A, the lumen pH decreased below 3.5 in stage 9B. An increase of HCl production capacity during larval development has previously been reported in several other teleost species [22,53-56]. However, the present study revealed that there was co-ordination between morphological changes and the key elements essential for the stomachs proteolytic activity such as H^+^/K^+^-ATPase and HCl production and the THs most likely orchestrate this change. During the climax of metamorphosis, a rapid colour change from acidic to alkaline was observed in the midgut when the pH indicator solution passed through the pyloric sphincter. Similar findings were reported for seabass (*Lates calcarifer*: [54]) and Japanese flounder [53]. This observation in Atlantic halibut at metamorphic climax suggests active secretion of ${\mathrm{HCO}}_{3}^{-}$ into the midgut, most likely via Cl^-^/${\mathrm{HCO}}_{3}^{-}$ exchange in the apical membrane of enterocytes [53,57-59], and also alkaline bile and liver secretions. Future studies will be required to determine whether THs trigger the events that lead to the development of a functional stomach in other altricial-gastric species as well.

Previous studies have shown that Atlantic halibut ghrelin was predominantly expressed in the stomach area but was also detected in pyloric caeca, immature gonads and intestine [33,34]. In newly hatched yolk-sac larvae, ghrelin protein was widely distributed in the GI-tract and was present in the anterior GI-tract before the gastric glands and *pepsinogen* production appeared [34]. In the present study *ghrelin* mRNA expression levels greatly increased during stomach differentiation in metamorphosis proclimax and climax. The significant correlation between *ghrelin* mRNA expression and *pepsinogen A2* and gastric proton pump subunit transcript expression appears to confirm the link between ghrelin and the acquisition of stomach proteolytic function. Intriguingly, in weanling pigs the physiological role of ghrelin in appetite stimulation has been correlated with the initiation of the stomachs proteolytic activity [60] and a similar association may also occur in halibut.

The presence of the pyloric sphincter from stage 3 onwards and its functional activity - to hold ingested food in the stomach - were observed in the 3D models and in the *in vivo* studies. This indicated a small storage function that was already established in the Atlantic halibut presumptive stomach during early developmental stages. Although before metamorphosis, this function was mainly assumed by the anterior midgut (mg1), which has a bulb-like shape with a much larger volume. The presence of a “physiological” sphincter (specific region with a strong muscular contracting activity in the lower part of the midgut loop - area “A”) in the mg1 of the GI-tract allowed it to assume a reservoir function or at least to delay the chyme transit so that sufficient mixing with bile and digestive enzymes from the pancreas can occur. The lack of a fully developed stomach at stage 6 to mix the ingested food may be functionally compensated by the strong peristaltic activity (anterograde/retrograde contractions) observed in the mg1, which contributes to the mechanical degradation of the ingested food. This supports earlier notions in zebrafish (a stomachless species), proposing that the retrograde contractions observed in the anterior part of the midgut generate a similar mechanical mixing as the stomach [27]. Considered in the context of a chemical reactor [61] the Atlantic halibut GI-tract changed from a plug-flow reactor (PFR) operating system, in which ingested food flowed continuously through the intestine to a continuous-flow stirred-tank reactor (CTSR), with food entering and exiting continuously through the reaction vessel (acid stomach). It will be insightful in the future to model halibut GI-tract function during development in order to identify when critical changes occur and the regulatory processes that control them.

## Conclusions

In conclusion, this study contributes to our understanding of how TH-driven metamorphosis affects the morphology and the function of the GI-tract. The remodelling of Atlantic halibut GI-tract, specifically the stomach development and volume growth, is linked to the surge of TH levels during the climax of metamorphosis, and the morphological modifications are connected with a set of functional changes. We show that the proteolytic activity in the stomach starts during the climax of metamorphosis with the synchronized expression of *pepsinogen A2* and both gastric proton pump subunit transcripts. This ensures pepsinogen activation and creates the optimum pH range for pepsin activity. Furthermore, we demonstrate that stomach ghrelin, a key element for the gastric involvement in appetite regulation, is correlated with the emergence of proteolytic activity. The presumptive stomach has a storage capacity in early development, however the main storage function is assumed by the anterior part of the midgut before metamorphic climax. During the metamorphic climax the main short term storage capacity shifts to the stomach, when its volume increases, and the GI-tract motility patterns change with a decrease in contractions of the midgut due to the functional development of the stomach. Considering the generally conserved nature of the post-embryonic modifications of the GI-tract in altricial-gastric species, our results are likely a general characteristic of teleost fish and potentially other vertebrates. However, further research is required to substantiate this general hypothesis and elucidate the molecular mechanisms regulating the functional development of the GI-tract.

## Methods

### Larvae and sampling

The material for the present study came from different batches of commercially produced Atlantic halibut larvae. Larvae used for 3D modeling were the same as previously described by Kamisaka et al. [62] except for the last developmental stage (stage 10), where complementary material was sampled at Nordic Halibut (Askøy, Norway). For all other analysis, larvae were sampled at Sterling White Halibut AS (Marine harvest, Rørvik, Norway) during March 2012. Larvae were reared according to standard industrial protocols, with light/dark cycles of 18:6 hours and water temperature 11°C. Feeding with *Artemia* enriched with commercial products took place twice a day (10:00 and 22:00) following standard rearing procedures [63].

Classification of developmental stage was based on mytome height (MH) and standard length (SL), according to a modified version of Sæle et al. [64]. The following stages were used in the functional studies: 5 - premetamorphic; 6 and 7 - prometamorphic; 8 - proclimax metamorphosis; 9A and 9B - climax metamorphosis; and 10 - post-metamorphosis. For the morphological studies (3D models) two extra stages were included, stage 3 and 4, based on morphological classification criteria of Pittman et al. [25]. Larvae intended for gene expression analysis were sampled 2 h after feeding (12:00) and euthanized with a lethal dose of MS222 (Tricaine methanesulfonate, Sigma-Aldrich, St. Louis, USA). Photos of each larva were taken in order to categorise them into different developmental stages. The GI-tract from each larva was dissected, rapidly transferred to RNAlater (Life Technologies, Carlsbad, USA) and stored at − 80°C. Atlantic halibut larvae used for *in vivo* studies (pH and motility analysis) were staged based on the photographs of living individuals.

To clone and study the expression profiles of p*epsinogen*, *H*^*+*^*/K*^*+*^*-ATPase α* and *β subunit*, *Na*^*+*^*/K*^*+*^*-ATPase subunit α* and *ghrelin*, Atlantic halibut juveniles (147.7 ± 15.1 g wet weight; 23.4 ± 1.1 cm total length; *n* = 6) were sampled at the Institute of Marine Research, Austevoll, Norway. The fish were euthanized with a lethal dose of MS222. The GI-tract was dissected into stomach, pyloric caeca, midgut and hindgut and stored in RNAlater at − 80°C until further analysis.

The experimental procedures and sampling protocols in the study were approved by an ethical committee (No. 2679; IMR Austevoll, Norway). All procedures were performed by scientists licensed by the Norwegian Animal Research Authority (NARA) to work on animals and under due consideration of the NARA guidelines.

### 3D reconstruction of digestive organs

For reconstruction of the digestive organs, six high quality preserved larvae were used for each stage studied (stages 3, 4, 5, 6, 9A and 10) and then the most representative larvae from each stage was used to construct the 3D model. Detailed material information about the approach taken is given in [62]. In summary, sampled larvae were fixed in Bouin’s solution overnight, stored in 70% EtOH at 4°C, dehydrated and embedded in paraffin. Serial sections were cut at 5 μm thickness and counterstained with hematoxylin. For the oldest stage, halibut larvae were fixed in 4% paraformaldehyde, dehydrated through an ethanol series and embedded in Technovit 7100 (Heraeus Kulzer GmbH, Hanau, Germany). Semi-thin (2 μm) serial sections were stained with Toluidin blue.

Photographs were taken every fifth section (10 μm between used sections) using a Nikon Digital Sight DS-U1 camera mounted on a Zeiss Axioscope 2 Plus microscope. The 3D reconstruction of the digestive system was performed as described by Kamisaka and Rønnestad [43]. In brief, manually defined contour lines of the digestive organs were made based on aligned images of serial sections, and contour surfaces were calculated using the software Imaris 6.2.0. (Bitplane AG Zurich, Switzerland). After generating a surface object, the same software (Imaris MeasurementPro) automatically calculated a range of statistical parameters including surface area and volume of the different organs. The volume increase of the digestive organs between stages was calculated and normalized to the overall mean of volume increase for each tissue (see Additional file 2).

### Cloning of pepsinogen, ghrelin, Na^+^/K^+^-ATPase subunit α and gastric proton pump subunits sequences

Total RNA was isolated from the GI-tract of juvenile Atlantic halibut using TRI reagent (Sigma-Aldrich, St. Louis, USA) according to the manufacturer’s instructions. Samples were treated with TURBO DNA-free (LifeTechnologies, Austin, USA) to eliminate genomic DNA contamination. Quality of DNase treated total RNA was assessed using an Agilent 2100 Bioanalyzer (Agilent Technologies). cDNA was synthesized from 2.0 μg of DNase treated total RNA using oligo (dT) primer from SuperScript III First-Strand Synthesis system for RT-PCR kit (Invitrogen, Carlsbad, USA).

Transcript fragments of *pepsinogen A2*, *ghrelin* [GenBank: EF493849], gastric proton pump subunits and *Na*^*+*^*/K*^*+*^*-ATPase subunit α* were amplified using gene specific primers as listed in Table 4 designed with Primer Premier 5 software (Premier Biosoft Int., Palo Alto, USA). For *pepsinogen A2* and *H*^*+*^*/K*^*+*^*-ATPase β subunit*, a PCR homology-cloning approach was used with primers designed in putative conserved N and C terminus regions of the winter flounder [GenBank: AF156788] and stickleback (*Gasterosteus aculeatus*, [Ensembl: ENSGACT00000020259]) homologue genes, respectively. The *H*^*+*^*/K*^*+*^*-ATPase α subunit* was cloned taking a comparative homology approach using the winter flounder homologue gene [GenBank: AF156789.1]. The *Na*^*+*^*/K*^*+*^*-ATPase α subunit* was cloned based on two ESTs from Atlantic halibut [GenBank: EB031798 and EB031117]. Amplifications were performed in a thermocycler Gene Amp PCR system 2700 (Applied Biosystems) using GoTaq DNA polymerase (Promega, Madison, USA) according to the manufacturer’s instructions and using the following conditions: 95°C for 2 min; 30 cycles of 95°C for 30 s, 58°C for 30 s, 72°C for 30 s; and a final step at 72°C for 5 min. Amplified PCR products were resolved on a 1% agarose gel and purified using E.Z.N.A. Gel Extraction Kit (Omega bio-tek, Norcross, USA). Purified fragments were cloned into the pGem-T easy vector system I (Promega, Madison, USA) and sequenced at the University of Bergen Sequencing Facility (Bergen, Norway). Sequence identity was confirmed by BLASTx (http://blast.ncbi.nlm.nih.gov/Blast.cgi) analysis against the GenBank database.

**Table 4:** Sequence of the specific primers used for cloning and qPCR gene expression analysis

	**Cloning**		**For quantitative PCR**	
**Gene**	**Primer**	**Sequence (5′ → 3′)**	**Primer**	**Sequence (5′ → 3′)**
*Pepsinogen A2*				
	PepA2-F	ATGAAGTGGCTCGTTGTTCTCT	PepA2-qF	TACGATGCCAACCACTTCA
	PepA2-R	TTACACGGACTTGGCCAGACCAATG	PepA2-qR	GATGGGCCAGCGATCAGGGAG
*H*^*+*^*/K*^*+*^*-ATPase α subunit*				
	HKA-F	GTCTGGACTGTGCTTTGCT	HKA-qF	AGCCAATGTTGGCATCATCTCA
	HKA-R	CGCACAACAGCGGGAACCAG	HKA-qR	CGTCATCCAACTCCTCACT
*H*^*+*^*/K*^*+*^*-ATPase β subunit*				
	HKB-F	ATGGCCGCCTTGAAGGAGAA	HKB-qF	GGAGAAGAGGACCTGTGG
	HKB-R	TTATTTCACTGCTTTCAGGGAA	HKB-qR	AGAACGCCAAGTAATACAA
*Ghrelin*				
	Ghr-F	TTAACACTCTATGTCCCTTCATCA	Ghr-qF	GGCTGCTGGTTGTTCTACTCTG
	Ghr-R	GTCAGTTGATGCTTTATTTTTACCACC	Ghr-qR	TCCTCGGTGGGTTGATTCTG
*Na*^*+*^*/K*^*+*^*-ATPase α subunit*				
	NaKA-F	CTGAAGGCAACGAGACTGT	NaKA-qF	CTGAAGGCAACGAGACTGT
	NaKA-R	GGATGACGAAATATGTGAAGAA	NaKA-qR	CGAGGTTCTGGCGAAGACGAT
*Elongation factor 1 alpha*				
			EF-qF	CGAGAAGTTCGAGAAGGAAGCT
			EF-qR	ACCCAGGCGTACTTGAAGGA

### Sequence comparisons and phylogenetic analysis

Multiple sequence alignments of H^+^/K^+^-ATPase subunit α and β and Na^+^/K^+^-ATPase subunit α protein sequence were performed with ClustalX (Gonnet 250 series matrix, Gap opening penalty 10, Gap extension 0.2) [65]. Alignments were displayed in GeneDoc (http://www.nrbsc.org/gfx/genedoc/) and percentage of sequence identity and similarity calculated. Phylogenetic analyses were performed using the Maximum Likelihood method [66] with 1000 bootstrap replicates [67], using MEGA 5.2 software [68].

### Quantitative real-time PCR assays

Total RNA was isolated from the GI-tract of the larvae at each developmental stage and cDNA synthesized as described above. For expression pattern analysis, specific primers were designed for the target genes (Table 4) and the target amplified using a Bio-Rad CFX96™ Real-Time System. The gene eEF1AI (Elongation factor 1 alpha, [GenBank: EU561357]) was used as the internal reference gene [69]. Relative gene quantification was performed using the mean normalized expression (MNE) method of the Q-Gene application [70,71]. Assay efficiency was determined using a 10-fold cDNA pool dilution curve ranging from 200 to 0.02 ng. Reactions for each sample were performed in duplicated using the following PCR conditions: 95°C for 3 min; 45 cycles of 95°C for 30 sec, 58°C for 30 sec and 72°C for 30 sec. Melting curve analysis over a range of 45-95°C (increment of 0.5°C for 4 sec) allowed the detection of nonspecific products and/or primer dimers.

The mRNA expression levels are presented as the mean ± SEM (n = 6). Data was log-transformed to achieve normal distribution. Statistical significance of relative gene expression between groups was analysed by one-way ANOVA when the data-set had a normal distribution. One-way ANOVA followed by a Student-Newman-Keuls (SNK) multiple range test was applied when data failed the normality test. SigmaStat v.3.1 (Systat software, Inc., USA) was used for the statistical analysis.

Correlation analysis were performed between: A) *H*^*+*^*/K*^*+*^*-ATPase α subunit* versus *β subunit*; B) *pepsinogen A2* versus gastric *H*^*+*^*/K*^*+*^*-ATPase α* and *β subunits*; C) *ghrelin* versus *pepsinogen A2* and gastric *H*^*+*^*/K*^*+*^*-ATPase α* and *β subunits*. Assuming that the relationship is linear, a linear model (lm) [72,73] was applied to the mean of the log-transformed MNE of the transcripts through development (stage 5 to 10). Plot graphs were constructed based on the linear model results. The correlation analysis were conducted in R [74].

### Assessment of pH in the stomach lumen and detection of acid production

The pH in the lumen of the GI-tract was determined with an *in vivo* method where pH indicator solutions (from alkaline to acidic ranges) were administered by tube feeding [53]. The *in vivo* set-up comprised a stereo dissecting microscope with a Leica DFC295 camera and a micromanipulator. A nanoliter injector (World Precision Instruments) with a plastic capillary tube (O.D. 0.19 mm, Sigma-Aldrich, St. Louis, USA) was fastened to the micromanipulator. The larvae were anaesthetized (MS-222; ranging from 3 to 100 μg/ml final concentration) and placed on a microscope slide in a droplet of clean seawater. The correct position of the larvae for injection was assured by the water surface tension. The capillary tube was gently passed through the mouth and esophagus into the presumptive stomach/stomach area and one of three pH indicator solutions was injected into a total of 3 larvae at each developmental stage (Figure 6). The first solution (*sol* mCP) consisted of 0.1% m-Cresol purple (Sigma-Aldrich, St. Louis, USA) in sea water (pH range 7.5–9.5). The second solution (*sol* CPR) contained 0.1% of Chlorophenol Red (Sigma-Aldrich, St. Louis, USA) in sea water (pH range 6–9.5) and the third (*sol* BPB) was 1% Bromophenol Blue (Sigma-Aldrich, St. Louis, USA) in sea water (pH range 3.0-4.6). The colour of the intestinal fluid was then compared to a set of solutions mCP, CPR and BPB standards prepared in pH buffers from pH 2.0 to pH 9.0 in steps of 0.5. The standards were inside sealed glass capillaries and immersed in sea water with the same light and temperature conditions as the larvae under the dissecting microscope. All experimental fish were euthanized with an overdose of MS222 after treatment. The work with Atlantic halibut larvae was conducted in a cold room to ensure a constant temperature.

### Motion analysis of GI-tract motility

Analysis of the GI-tract motility pattern in halibut larvae was based on *in vivo* video recordings, using a Leica DFC295 camera connected to a stereo dissecting microscope. Video sequences were recorded from four larvae with a full or partially full GI-tract at two different developmental stages: stage 6 - prometamorphic and stage 9 - climax metamorphosis. The animals were anaesthetized and maintained immersed in seawater on a microscopic slide and captured on video for 30 min. From the video recordings, still images were captured for analysis and identification of the different motility patterns. The GI-tract movements were quantified using Etholog 2.2 software [75]. The number and frequency (number of contractions min^-1^) of phasic and propagating wave contractions in two different regions of the midgut were determined. The frequency and number of contractions in the presumptive stomach/stomach area, hindgut and in the rectal area were also quantified.

### Availability of supporting data

All supporting data are included in the Additional files. In addition, nucleotide sequences have been deposited in GenBank under the accession numbers: KF184647 (pepsinogen A2); KF184648 (H^+^/K^+^-ATPase α subunit); KF184650 (Na^+^/K^+^-ATPase α subunit); KF184649 (H^+^/K^+^-ATPase β subunit) and the alignments for the phylogenetic tree construction are available in TreeBase: http://purl.org/phylo/treebase/phylows/study/TB2:S15435.

## Competing interest

The authors declare that they have no competing interest.

## Authors’ contributions

ASG and IR designed the study. ASG carried out the analyses and drafted the manuscript. YK preformed the 3D model and TH supported the sampling and the *in vivo* experiments. ASG, DMP, YK and IR contributed to interpretation of the data. All authors contributed to the writing of the manuscript, read and approved the final version.

## Supplementary Material

Additional file 1 **Stomach histology of Atlantic halibut juvenile at stage 10 (65 dpff).** es: esophagus, gg: gastric gland, hg: hindgut, li: liver, mg: midgut, pc: pyloric caeca, ps: pyloric sphincter, st: stomach. Click here for file

Additional file 2 Digestive organ volume increase between stages and normalization to the overall mean of volume increase. Click here for file

Additional file 3 **Nucleotide and deduced amino acid sequences of Atlantic halibut H**^**+**^**/K**^**+**^**-ATPase β subunit (A); H**^**+**^**/K**^+^**-ATPase α subunit (B); Pepsinogen A2 (C) and Na**^**+**^**/K**^**+**^**-ATPase α subunit (D).** Numbers on the right refer to the positions of nucleotides (upper row) and amino acids (lower row). White and grey boxes indicate primer regions for qPCR analysis and cloning, respectively. Predicted intron/exon borders are represented with black triangles. In (C) the pro-segment is underlined with the signal peptide preceding it. Cysteine residues involved in disulfide bonds (C), the putative active site Asp (D) in (C) and regions containing N-glycosylation sites (N) in (A) are indicated. The amino acids involved in metal (magnesium) binding are marked with a white circle. Click here for file

Additional file 4 **Evolutionary analysis of H**^**+**^**/K**^**+**^**-ATPase and Na**^**+**^**/K**^**+**^**-ATPase α subunit among vertebrates using the Maximum Likelihood method based on the JTT matrix-based model (1000 bootstrap replicates) with MEGA5.2 software.** The tree with the highest log likelihood (−1943.1218) is shown. The scale bar indicates the substitution rate per residue. NCBI or Ensembl sequence accession numbers are shown after the common species name. Click here for file

Additional file 5 **Evolutionary analysis of H**^**+**^**/K**^**+**^**-ATPase and Na**^**+**^**/K**^**+**^**-ATPase β subunit precursor among vertebrates using the Maximum Likelihood method (1000 bootstraps replicates, JTT matrix-based model) with MEGA5.2 software.** The tree with the highest log likelihood (−5184.5314) is shown. The scale bar indicates the substitution rate per residue. NCBI or Ensembl sequence accession numbers are shown after the common specie name. Click here for file

Additional file 6 **Still images extracted every 10 sec for a total period of 30 seconds from video records, illustrating the constant contraction state of Atlantic halibut larva 4, stage 6 (see Table **3**).** The red arrow indicates the point of muscle contraction in midgut region 1 (mg1) and the white arrow in midgut region 2 (mg2). Click here for file

Additional file 7 **Still images extracted from video records illustrating the different motility patterns in the stomach (A); midgut region 1 (B); midgut region 2 and hindgut (C) of Atlantic halibut larvae.** The arrows indicate the point of muscle contraction. For propagating waves, the first point of contraction is marked in all pictures by a dashed line to follow the wave movement. Click here for file
