# Supplementary figures and images for: Functional modifications associated with gastrointestinal tract organogenesis during metamorphosis in Atlantic halibut (Hippoglossus hippoglossus)
Source: BMC Dev Biol. 2014 Feb 19;14:11. doi: 10.1186/1471-213X-14-11 (PMC3940299; doi:10.1186/1471-213X-14-11)

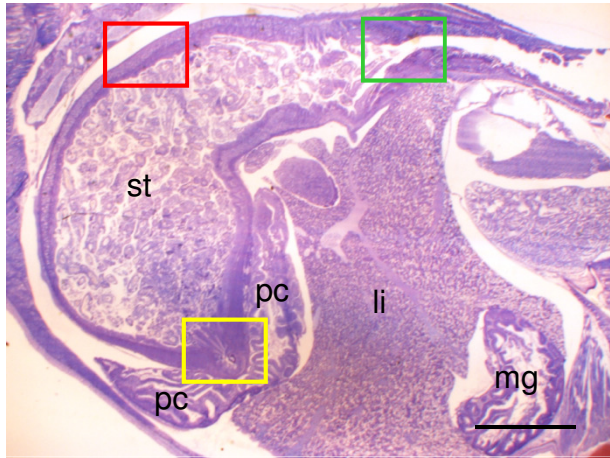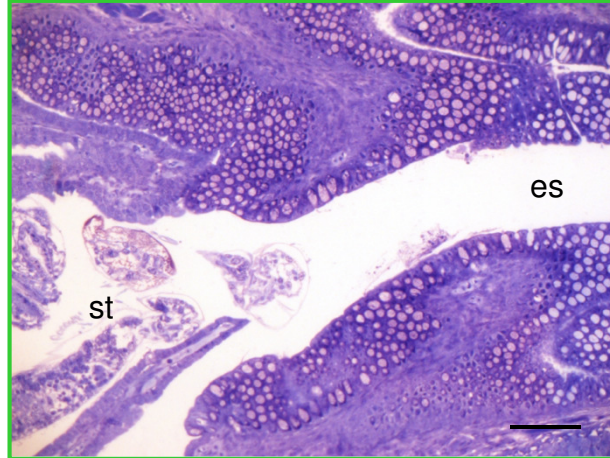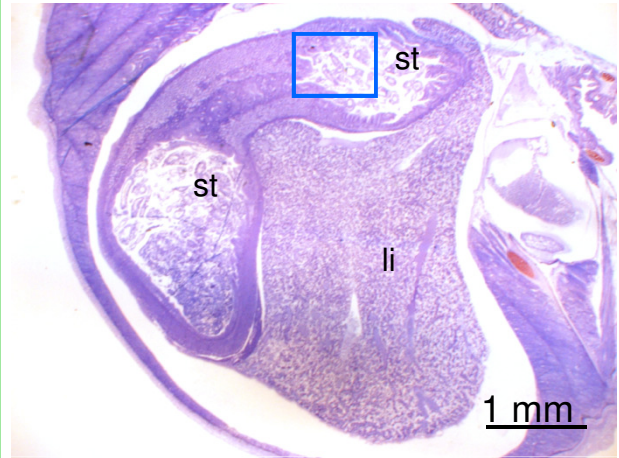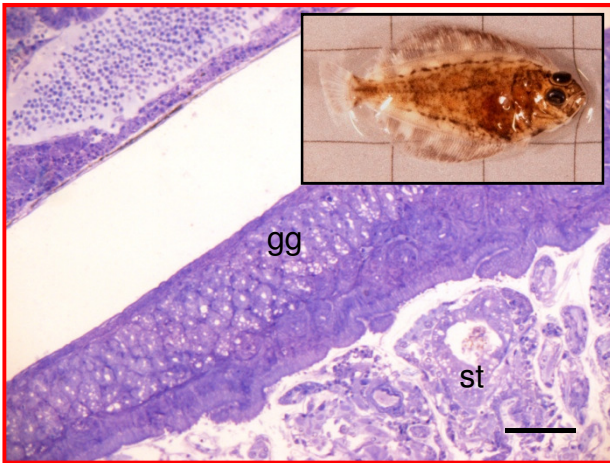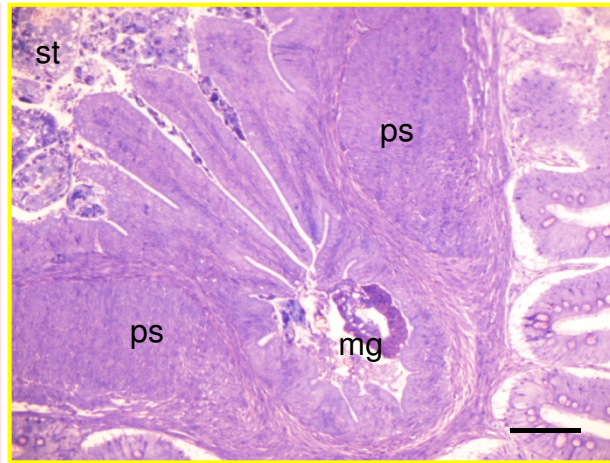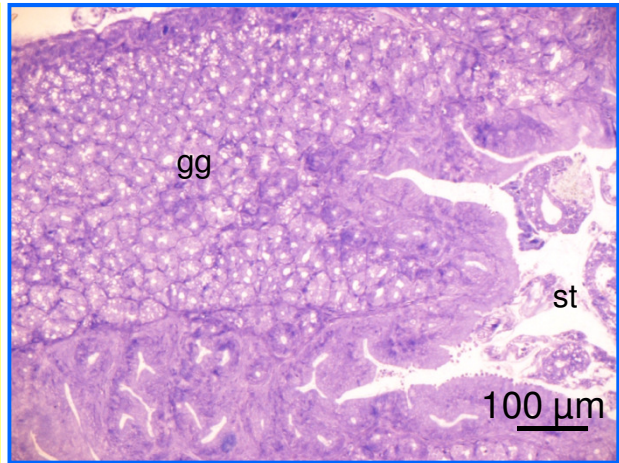

Supplement: Additional file 1 — Stomach histology of Atlantic halibut juvenile at stage 10 (65 dpff). es: esophagus, gg: gastric gland, hg: hindgut, li: liver, mg: midgut, pc: pyloric caeca, ps: pyloric sphincter, st: stomach. [file 1471-213X-14-11-S1.pdf]

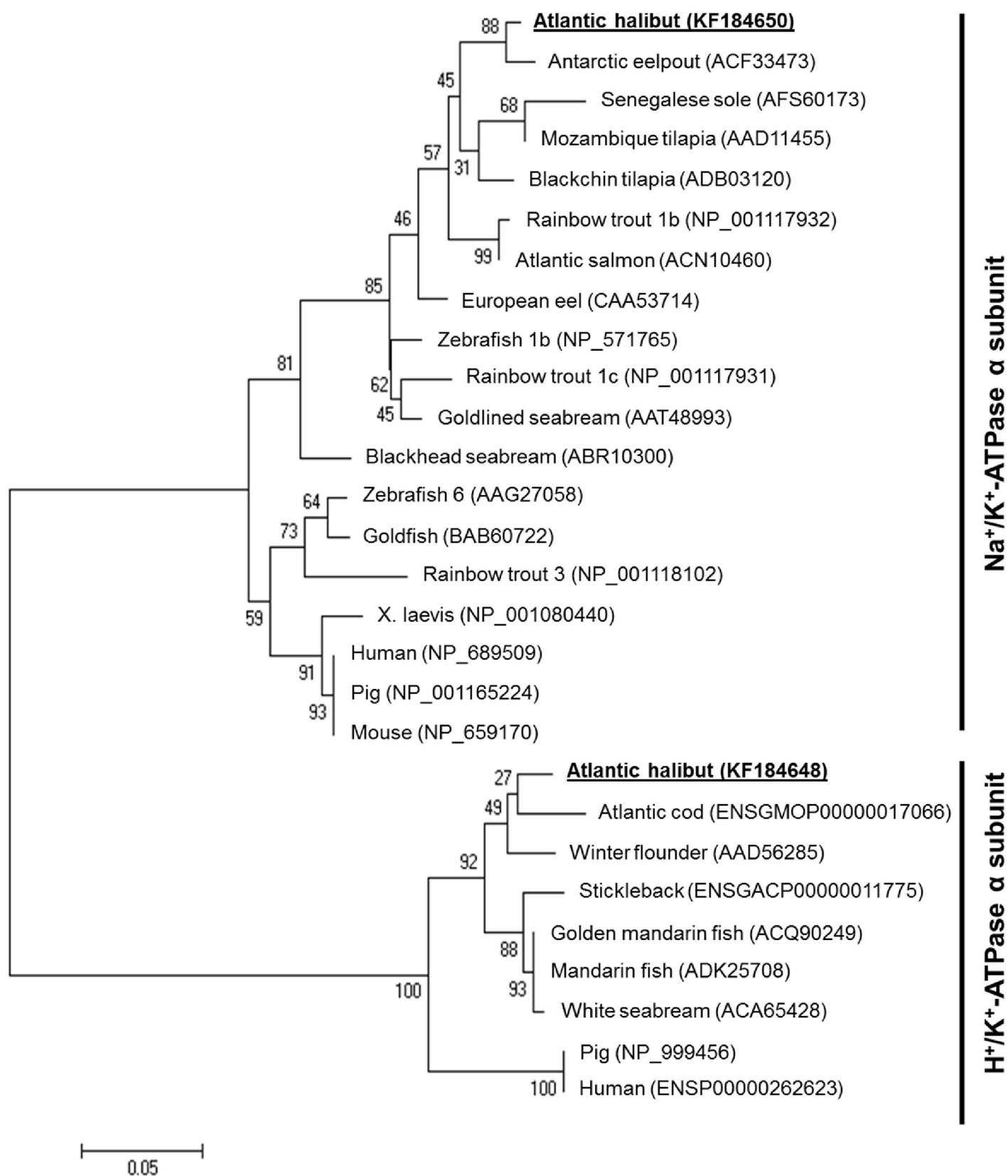

Supplement: Additional file 4 — Evolutionary analysis of H+/K+-ATPase and Na+/K+-ATPase α subunit among vertebrates using the Maximum Likelihood method based on the JTT matrix-based model (1000 bootstrap replicates) with MEGA5.2 software. The tree with the highest log likelihood (−1943.1218) is shown. The scale bar indicates the substitution rate per residue. NCBI or Ensembl sequence accession numbers are shown after the common species name. [file 1471-213X-14-11-S4.pdf]

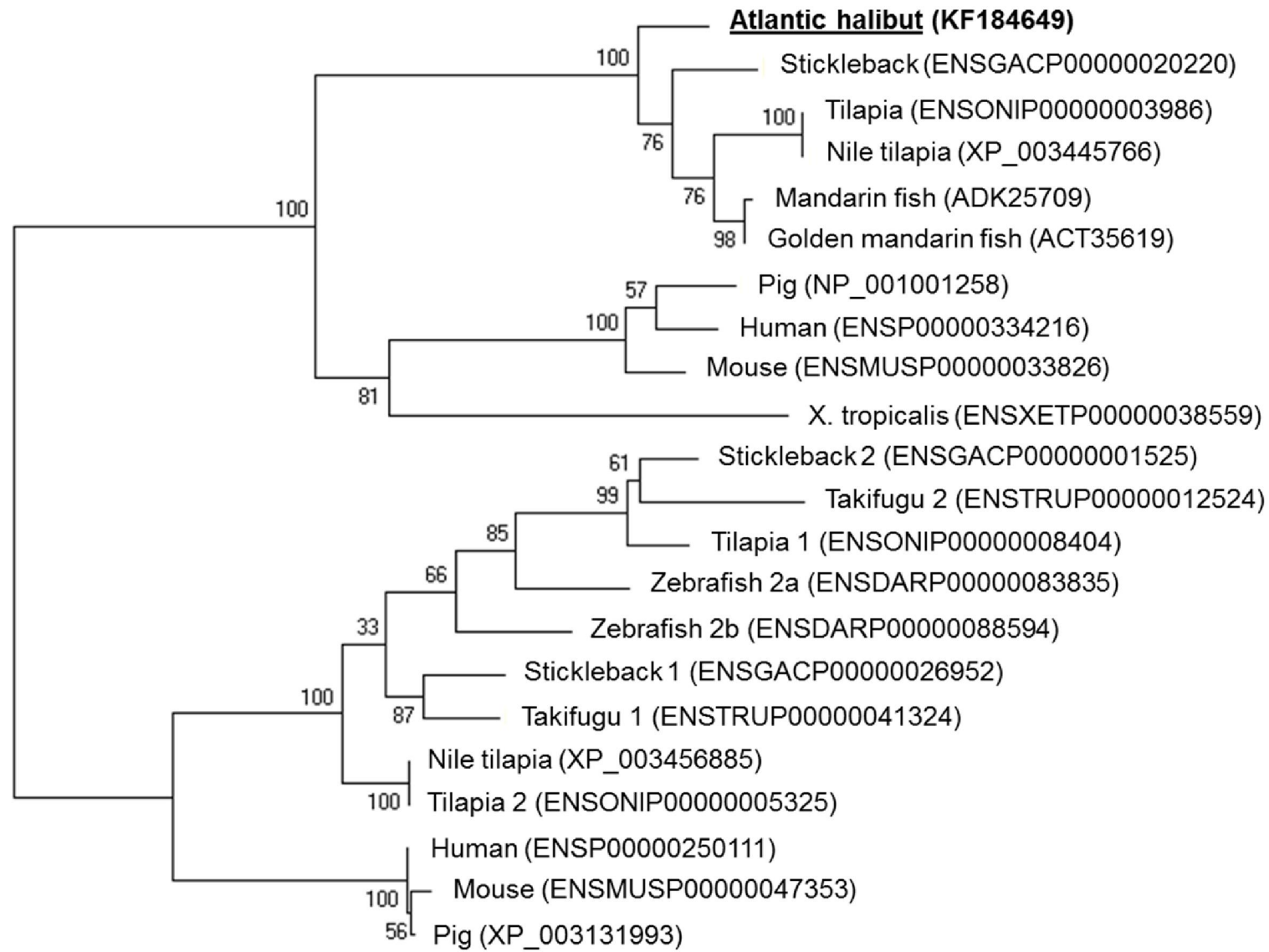

**Na<sup>+</sup>/K<sup>+</sup>-ATPase β subunit**      **H<sup>+</sup>/K<sup>+</sup>-ATPase β subunit**

Supplement: Additional file 5 — Evolutionary analysis of H+/K+-ATPase and Na+/K+-ATPase β subunit precursor among vertebrates using the Maximum Likelihood method (1000 bootstraps replicates, JTT matrix-based model) with MEGA5.2 software. The tree with the highest log likelihood (−5184.5314) is shown. The scale bar indicates the substitution rate per residue. NCBI or Ensembl sequence accession numbers are shown after the common specie name. [file 1471-213X-14-11-S5.pdf]

## Larva 4 – Stage 6

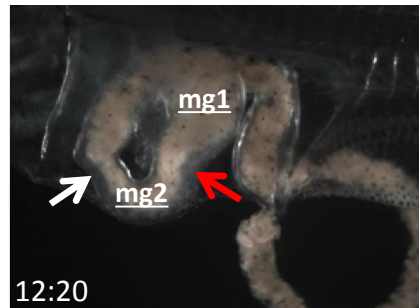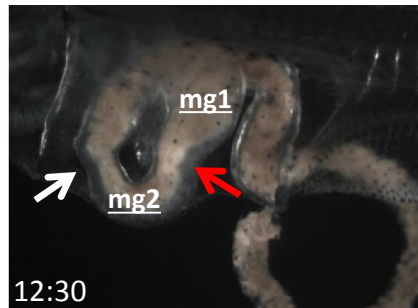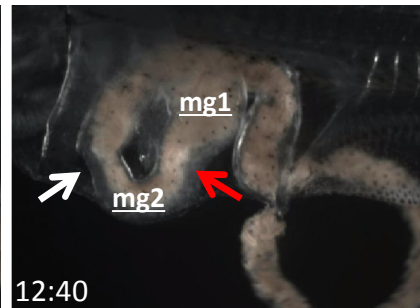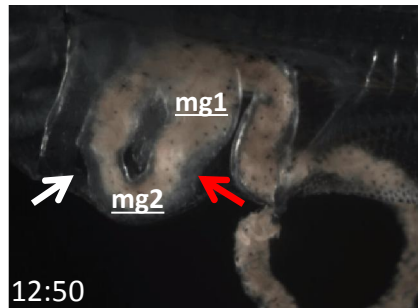

Supplement: Additional file 7 — Still images extracted from video records illustrating the different motility patterns in the stomach (A); midgut region 1 (B); midgut region 2 and hindgut (C) of Atlantic halibut larvae. The arrows indicate the point of muscle contraction. For propagating waves, the first point of contraction is marked in all pictures by a dashed line to follow the wave movement. [file 1471-213X-14-11-S7.pdf]
