# Supplementary material for: Functional modifications associated with gastrointestinal tract organogenesis during metamorphosis in Atlantic halibut (Hippoglossus hippoglossus)
Source: BMC Dev Biol. 2014 Feb 19;14:11. doi: 10.1186/1471-213X-14-11 (PMC3940299; doi:10.1186/1471-213X-14-11)
Supplement: Additional file 2 — Digestive organ volume increase between stages and normalization to the overall mean of volume increase. [file 1471-213X-14-11-S2.pdf]

Supplemental table 1: Digestive organs volume increase between stages (S). The increase volume were calculated with: $Si \rightarrow Sf = \left(\frac{Sf}{Si} \times 100\right) - 100$, Si is the initial stage and Sf the final stage.

	S3->S4	S4->S5	S5->S6	S6->S9A	S8->S10	Mean
GI-tract outer layer	65.82	87.97	111.65	157.12	381.44	160,80
GI-tract inner layer	42.58	31.67	97.30	96.64	523.84	158,41
GI-tract tissue[a]	74.55	94.78	128.73	219.17	291.43	161,73
Liver	67.30	102.76	129.41	312.13	355.99	193,52
Pancreas	57.16	80.46	187.73	332.02	-1.58	131,16
Islets of Langerhans	38.53	101.12	84.21	407.62	120.08	150,31
Presumptive stomach	55.33	76.98	162.29	392.97	1089.91	355,50
					**Overall mean**	**187.35**

[a] GI-tract tissue volume = GI-tract outer layer - GI-tract inner layer

Supplemental table 2: Standardized digestive organs volume increase. The volume increase (see supplemental table 1) was normalized to the overall mean of volume increase between stages for each tissue.

	S3->S4	S4->S5	S5->S6	S6->S8	S8->S10
GI-tract outer layer	0.35	0.47	0.60	0.84	2.04
GI-tract inner layer	0.23	0.17	0.52	0.52	2.80
GI-tract tissue[a]	0.40	0.51	0.69	1.17	1.56
Liver	0.36	0.55	0.69	1.67	1.90
Pancreas	0.31	0.43	1.00	1.77	-0.01
Islets of Langerhans	0.21	0.54	0.45	2.18	0.64
Presumptive stomach	0.30	0.41	0.87	2.10	5.82

[a] GI-tract tissue volume = GI-tract outer layer - GI-tract inner layer
