# Supplementary material for: Functional modifications associated with gastrointestinal tract organogenesis during metamorphosis in Atlantic halibut (Hippoglossus hippoglossus)
Source: BMC Dev Biol. 2014 Feb 19;14:11. doi: 10.1186/1471-213X-14-11 (PMC3940299; doi:10.1186/1471-213X-14-11)
Supplement: Additional file 3 — Nucleotide and deduced amino acid sequences of Atlantic halibut H+/K+-ATPase β subunit (A); H+/K+-ATPase α subunit (B); Pepsinogen A2 (C) and Na+/K+-ATPase α subunit (D). Numbers on the right refer to the positions of nucleotides (upper row) and amino acids (lower row). White and grey boxes indicate primer regions for qPCR analysis and cloning, respectively. Predicted intron/exon borders are represented with black triangles. In (C) the pro-segment is underlined with the signal peptide preceding it. Cysteine residues involved in disulfide bonds (C), the putative active site Asp (D) in (C) and regions containing N-glycosylation sites (N) in (A) are indicated. The amino acids involved in metal (magnesium) binding are marked with a white circle. [file 1471-213X-14-11-S3.pdf]

(A) *H^+/K^+-ATPase β subunit*

```
                HKB-F ──►   HKB-qF ──►
atggccgccttgaaggagaagaggacctgtgcgcagcgatgtgaagactttggccgtttt 60
 M  A  A  L  K  E  K  R  T  C  G  Q  R  C  E  D  F  G  R  F  20

gtctggaactcagacaatgggacatttatggggaggacaccagtgaaatgggtgtacatc 120
 V  W  N  S  D  N  G  T  F  M  G  R  T  P  V  K  W  V  Y  I  40
   ◄── HKB-qR
agcttgtattacttggcgttctacgtggtgatgacgggcctttttgtttggccatctat 180
 S  L  Y  Y  L  A  F  Y  V  V  M  T  G  L  F  C  L  A  I  Y  60

gtgctcatgtacaccttggatccgtacgcgcctgactaccaagaccggctaaaatcgcca 240
 V  L  M  Y  T  L  D  P  Y  A  P  D  Y  Q  D  R  L  K  S  P  80

ggggtgatggtgtggccggacacttatggagaggagaaagttgaaatcaactacaacaca 300
 G  V  M  V  W  P  D  T  Y  G  E  E  K  V  E  I  N  Y  N  T  100

tcagacaagtgcagctggatgaagatgaccaacatccttaataagttcctggagccctat 360
 S  D  K  C  S  W  M  K  M  T  N  I  L  N  K  F  L  E  P  Y  120

aatgacactgcacaacacgaatgttacaaccataactgcaccaagggaaagtactacatc 420
 N  D  T  A  Q  H  E  C  Y  N  H  N  C  T  K  G  K  Y  Y  I  140

cagaacaccttctctgcccctgaccacacaaagtgggcatgttccttcaccaaaagcatg 480
 Q  N  T  F  S  A  P  D  H  T  K  W  A  C  S  F  T  K  S  M  160

ctgggaccctgctcaggacacgaggacccgacctttggctacaactgcagcatgccgtgt 540
 L  G  P  C  S  G  H  E  D  P  T  F  G  Y  N  C  S  M  P  C  180

gtcgtgattaagatgaacaggatcatcaactttttgccaactaatgaaactgagcctccc 600
 V  V  I  K  M  N  R  I  I  N  F  L  P  T  N  E  T  E  P  P  200

ccatatgtcaactgtacgatactggaaggacatgacaatgtggacaaaatggagtatttc 660
 P  Y  V  N  C  T  I  L  E  G  H  D  N  V  D  K  M  E  Y  F  220

cctgaaggggggattatggatcgctcctactttccttattatggaaaacaggcacagcct 720
 P  E  G  G  I  M  D  R  S  Y  F  P  Y  Y  G  K  Q  A  Q  P  240

atgtatgtcaacccgttggtggccgttcgtttccacctggtcggagagaggtcggccaag 780
 M  Y  V  N  P  L  V  A  V  R  F  H  L  V  G  E  R  S  A  K  260

atccagtgcagagtggtctcccaccagatcagctaccaaaactaccacgacccctacgag 840
 I  Q  C  R  V  V  S  H  Q  I  S  Y  Q  N  Y  H  D  P  Y  E  280
               ◄── HKB-R
ggaaaagtcgactttttccctgaaagcagtgaaataa 876
 G  K  V  D  F  S  L  K  A  V  K  -  291
```

(B) *H^+/K^+-ATPase α subunit*

```
                HKA-F──►
gtctggactgtgctttgctggtctcatctcgatgattgacccccgagagccaccgtaccc 61
  S  G  L  C  F  A  G  L  I  S  M  I  D  P  P  R  A  T  V  P 20

gacgcagtgatgaaatgtcgtactgctggcatccgggtcgtcatggtgactggagaccat 121
 D  A  V  M  K  C  R  T  A  G  I  R  V  V  M  V  T  G  D  H  40
                                  HKA-qF──►
ccaatcacagcgagggcaatcgcagccaatgttggcatcatctcagaaggcagtgaaaca 181
 P  I  T  A  R  A  I  A  A  N  V  G  I  I  S  E  G  S  E  T  60

gtggaagatatcgcccagaggaagcgcattcctgtggaacaagtcaacaagagggacgcc 241
 V  E  D  I  A  Q  R  K  R  I  P  V  E  Q  V  N  K  R  D  A  80
                                           ◄──HKA-qR
cgtgcttgtgtgatcagcggtggtcagctgaaagacatgagcagtgaggagttggatgac 301
 R  A  C  V  I  S  G  G  Q  L  K  D  M  S  S  E  E  L  D  D  100

gccctgaggaaccaccccgagatggtgtttgcaagaacctcacctcagcagaaactgatc 361
 A  L  R  N  H  P  E  M  V  F  A  R  T  S  P  Q  Q  K  L  I  120

attgtggagagctgtcagcgtctgggctccatcgtcgctgttacaggtgatggagtgaac 421
 I  V  E  S  C  Q  R  L  G  S  I  V  A  V  T  G  D  G  V  N  140

gactcgccggctctcaagaaggccgacattggcattgccatggggatcgccggttcagat 481
 D  S  P  A  L  K  K  A  D  I  G  I  A  M  G  I  A  G  S  D  160

gctgccaagaacgctgctgacatgatcctgttggacgacaacttcgcttccatcgtcacc 541
 A  A  K  N  A  A  D  M  I  L  L  D  D  N  F  A  S  I  V  T  180

ggagtggagcagggccgtcttatctttgacaacctgaagaagtctatcgcctacacgctg 601
 G  V  E  Q  G  R  L  I  F  D  N  L  K  K  S  I  A  Y  T  L  200

accaaaaatattccggagctgactccatgtctggtctacatcaccgtcagcgtgcctctt 661
 T  K  N  I  P  E  L  T  P  C  L  V  Y  I  T  V  S  V  P  L  220

cctctcggctgcatcactatcctcttcatcgaactggccactgacatctacccctctgtc 721
 P  L  G  C  I  T  I  L  F  I  E  L  A  T  D  I  Y  P  S  V  240

tctctggcctatgagaaagcagagagtgacatcatgaacctgaagcccaggaaccctcgt 781
 S  L  A  Y  E  K  A  E  S  D  I  M  N  L  K  P  R  N  P  R  260

cgtgataggctggtaaacgaacccctggctgcgtactcgtactttcagataggagccatc 841
 R  D  R  L  V  N  E  P  L  A  A  Y  S  Y  F  Q  I  G  A  I  280
                                                   ◄──HKA-R
cagtcttttgcaggttttacagattatttctcagcgatggcccaggagggctggttcccg 901
 Q  S  F  A  G  F  T  D  Y  F  S  A  M  A  Q  E  G  W  F  P  300

ctgttgtgcg  911
 L  L  C    303
```

(C) *Pepsinogen A2:*

```
PepA2-F →
atgaagtggctcgttgttctctctgcccttgtggctttctccgagtgcctccataggatc 60
 M  K  W  L  V  V  L  S  A  L  V  A  F  S  E  C  L  H  R  I  20

ccccctgatcaaggggaagactgccaggcaagacctgcaggagaaaggactatgggaggag 120
 P  L  I  K  G  K  T  A  R  Q  D  L  Q  E  K  G  L  W  E  E  40

tacaggaaacagcacccatacaacccaatggccaagttcatccagagtggaactgagtcc 180
 Y  R  K  Q  H  P  Y  N  P  M  A  K  F  I  Q  S  G  T  E  S  60

atgactaatgatgctgacttgtcctactatggtgtgatctccatcggcacccctcctcag 240
 M  T  N  D  A  D  L  S  Y  Y  G  V  I  S  I  G  T  P  P  Q  80

tccttcaccgtcatctttgacacgggctcctccaacctgtgggtcccctccatctactgc 300
 S  F  T  V  I  F  D  T  G  S  S  N  L  W  V  P  S  I  Y  C  100

tccagccaggcctgtgacaaccacaagaaattcatcccccagcagtcctccaccttcaaa 360
 S  S  Q  A  C  D  N  H  K  K  F  I  P  Q  Q  S  S  T  F  K  120

tggggcagccagcctctgtccatccagtatggcactggcagcatgactggacgtctggcc 420
 W  G  S  Q  P  L  S  I  Q  Y  G  T  G  S  M  T  G  R  L  A  140

actgacactgttgaggtgggcggcctcactgtgaccaatcaggtgtttggaatcagccag 480
 T  D  T  V  E  V  G  G  L  T  V  T  N  Q  V  F  G  I  S  Q  160

acagaggctcccttcatggcccacatgaaggctgatggcatcctgggtctggccttccag 540
 T  E  A  P  F  M  A  H  M  K  A  D  G  I  L  G  L  A  F  Q  180

accatcgcttcggacaacgtcgtgcccgtcttcgataacatgatcagtcagaaccttgtg 600
 T  I  A  S  D  N  V  V  P  V  F  D  N  M  I  S  Q  N  L  V  200

tcccagcccatgttctccgtctacctgagcagcaaaagtcagcagggaagtgaggtggtc 660
 S  Q  P  M  F  S  V  Y  L  S  S  K  S  Q  Q  G  S  E  V  V  220
                 PepA2-qF →
ttcggtggttacgatgccaaccacttcactggtcaaatcagctggatccctctgacctct 720
 F  G  G  Y  D  A  N  H  F  T  G  Q  I  S  W  I  P  L  T  S  240

gccacctattggcagatcaagatggacagtgttaccatcaacggacagaccgtggcctgc 780
 A  T  Y  W  Q  I  K  M  D  S  V  T  I  N  G  Q  T  V  A  C  260
                                     ← PepA2-qR
tccggangctgccaggccatcatcgacaccggcacctccctgatcgctggcccatccagt 840
 S  G  X  C  Q  A  I  I  D  T  G  T  S  L  I  A  G  P  S  S  280

gacatcagcaacatgaattcctgggttggagcctcaaccaaccagtacggagaggctaca 900
 D  I  S  N  M  N  S  W  V  G  A  S  T  N  Q  Y  G  E  A  T  300

gtgaactgccagaacatccagagcatgcctgatgtcaccttcactctcgatggaaaggcc 960
 V  N  C  Q  N  I  Q  S  M  P  D  V  T  F  T  L  D  G  K  A  320

ttcaccatccccgcatctgcctacgtctctcagagctactatggttgcagcactggcttt 1020
 F  T  I  P  A  S  A  Y  V  S  Q  S  Y  Y  G  C  S  T  G  F  340

ggtcagggtggctctaacctctggatcctgggagacgtcttcatcagggagtactacgcc 1080
 G  Q  G  G  S  N  L  W  I  L  G  D  V  F  I  R  E  Y  Y  A  360
                         ← PepA2-R
gtctttgatgccccatccaagtacattggtctggccaagtccgtgtaa 1128
 V  F  D  A  P  S  K  Y  I  G  L  A  K  S  V  -  375
```

(D) *Na^+/K^+-ATPase α subunit*

```
NaKA-F / NaKA-qF──►
ctgaaggcaacgagactgttgaggacatcgctgcccgcctgaacgtcccagtgtcagaggtc 62
   E  G  N  E  T  V  E  D  I  A  A  R  L  N  V  P  V  S  E  V  20

aaccccagggatgccaaggcctgcgtcgtgcacggcggcgagctgaaagaaatgaccgag   122
 N  P  R  D  A  K  A  C  V  V  H  G  G  E  L  K  E  M  T  E    40
                                                  ◄── NaKA-qR
gagcacctcgacgatatcctgaagtaccacaccgagatcgtcttcgccagaacctcgcct   182
 E  H  L  D  D  I  L  K  Y  H  T  E  I  V  F  A  R  T  S  P    60

cagcagaagctgattattgtggagggttgccagagacagggcgccatcgtggccgtgaca   242
 Q  Q  K  L  I  I  V  E  G  C  Q  R  Q  G  A  I  V  A  V  T    80

ggtgacggcgtgaacgactctcctgctcttaaaaaggccgacatcggcgttgccatgggc   302
 G  D  G  V  N  D  S  P  A  L  K  K  A  D  I  G  V  A  M  G    100

atcgctggatctgacgtctccaagcaggccgctgacatgatcctgctggacgacaacttt   362
 I  A  G  S  D  V  S  K  Q  A  A  D  M  I  L  L  D  D  N  F    120

gcctccatcgttaccggcgtggaagaaggtcgtctgatctttgacaacttgaagaagtcc   422
 A  S  I  V  T  G  V  E  E  G  R  L  I  F  D  N  L  K  K  S    140

atcgcctacactctgaccagtaacatccccgagatctcacccttcctcttcttcatcatc   482
 I  A  Y  T  L  T  S  N  I  P  E  I  S  P  F  L  F  F  I  I    160

gccaacatccctctgcccctgggaaccgtcaccatcctctgtatcgacctgggaaccgac   542
 A  N  I  P  L  P  L  G  T  V  T  I  L  C  I  D  L  G  T  D    180

atggtccctgccatctccctggcttatgaagaagccgagagcgacatcatgaagaggcag   602
 M  V  P  A  I  S  L  A  Y  E  E  A  E  S  D  I  M  K  R  Q    200

cccagaaaccccaaaacggacaaactggtgaacgagaggctcatcagcatcgcctacgga   662
 P  R  N  P  K  T  D  K  L  V  N  E  R  L  I  S  I  A  Y  G    220
                                  ◄── NaKA-R
cagatcggtatgatgcaggccacagctggcttcttcacatatttcgtcatcc 714
 Q  I  G  M  M  Q  A  T  A  G  F  F  T  Y  F  V  I  237
```
