# Supplementary material for: Functional modifications associated with gastrointestinal tract organogenesis during metamorphosis in Atlantic halibut (Hippoglossus hippoglossus)
Source: BMC Dev Biol. 2014 Feb 19;14:11. doi: 10.1186/1471-213X-14-11 (PMC3940299; doi:10.1186/1471-213X-14-11)

A

Stomach

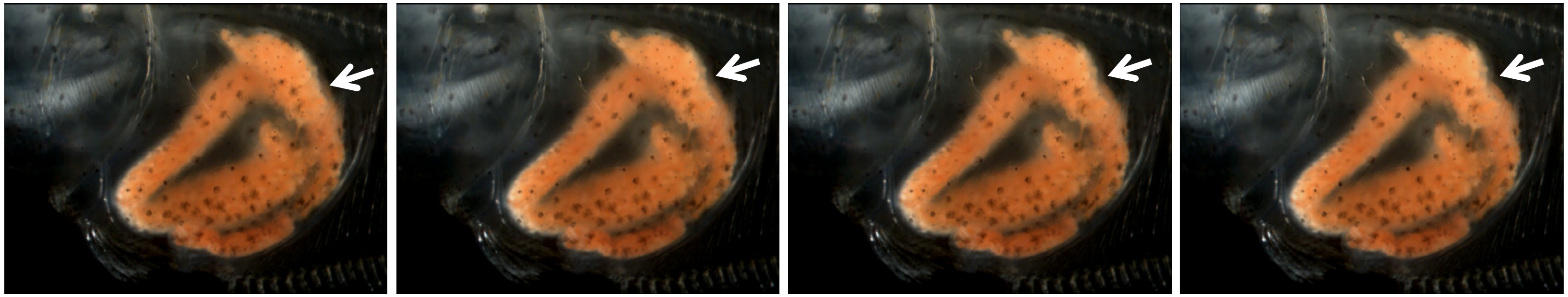

B

<u>Midgut region 1</u>

Propagating waves contraction:

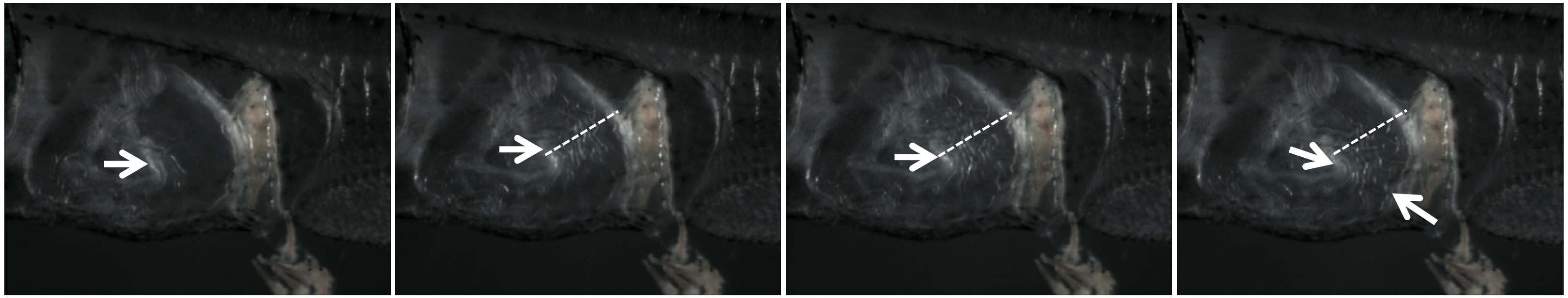

Phasic contraction:

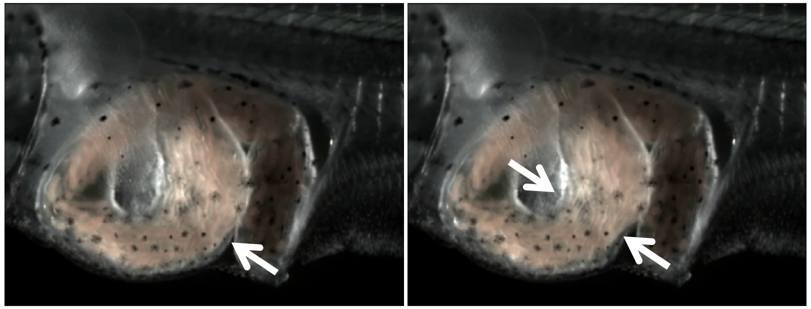

C

Midgut region 2 and Hindgut

Propagating waves contraction

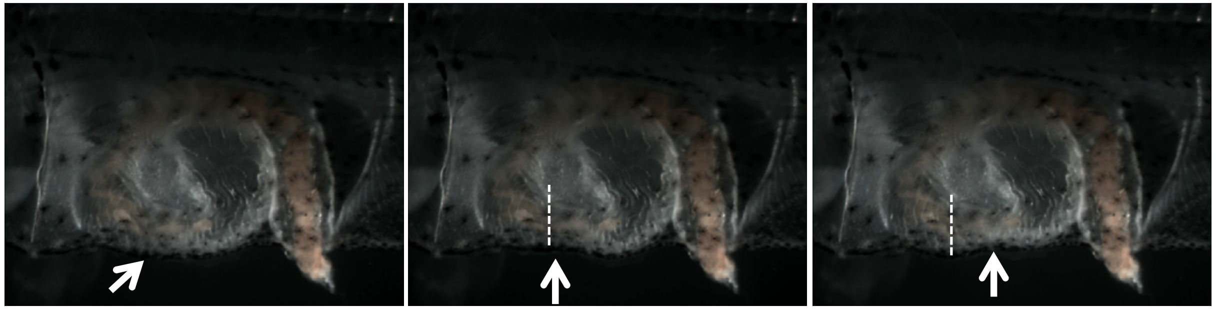

Phasiccontraction

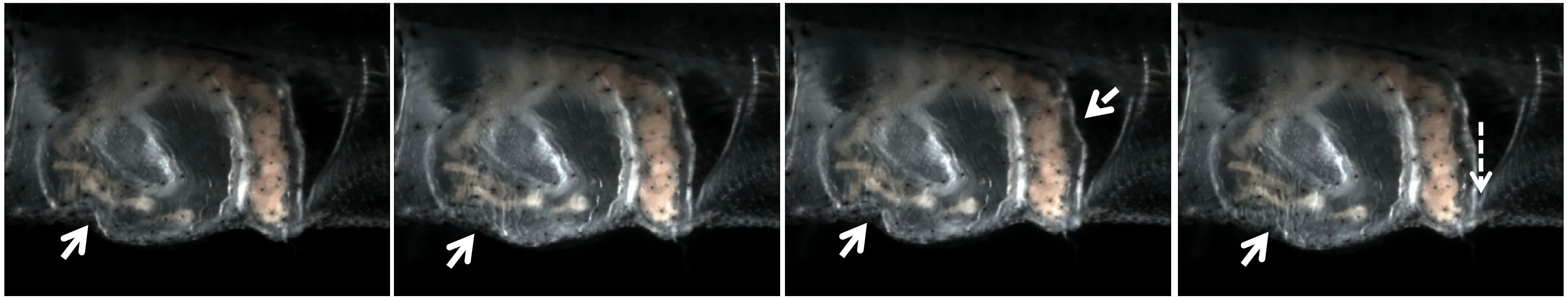

Supplement: Additional file 6 — Still images extracted every 10 sec for a total period of 30 seconds from video records, illustrating the constant contraction state of Atlantic halibut larva 4, stage 6 (see Table 3). The red arrow indicates the point of muscle contraction in midgut region 1 (mg1) and the white arrow in midgut region 2 (mg2). [file 1471-213X-14-11-S6.pdf]
